# Within-session dual-task walking practice improves gait variability in older adults with multiple sclerosis

**DOI:** 10.1016/j.gaitpost.2025.03.011

**Published:** 2025-03-19

**Authors:** Manuel E. Hernandez, Robert W. Motl, Frederick W. Foley, Meltem Izzetoglu, Mark Wagshul, Roee Holtzer

**Affiliations:** a Department of Biomedical and Translational Sciences, Carle Illinois College of Medicine, University of Illinois Urbana-Champaign, Urbana, IL, United States; b Department of Kinesiology and Community Health, College of Applied Health Sciences, University of Illinois Urbana-Champaign, Urbana, IL, United States; c Neuroscience Program, College of Liberal Arts & Sciences, University of Illinois Urbana-Champaign, Urbana, IL, United States; d Beckman Institute, University of Illinois Urbana-Champaign, Urbana, IL, United States; e Department of Kinesiology and Nutrition, College of Applied Health Sciences, University of Illinois Chicago, Chicago, IL, United States; f Ferkauf Graduate School of Psychology, Yeshiva University, Bronx, NY, United States; g Multiple Sclerosis Center, Holy Name Medical Center, Teaneck, NJ, United States; h Villanova University, Electrical and Computer Engineering, Villanova, PA, United States; i Department of Radiology, Gruss Magnetic Resonance Research Center, Albert Einstein College of Medicine and Montefiore Medical Center, Bronx, NY, United States; j Department of Neurology, Albert Einstein College of Medicine, Bronx, NY, United States

**Keywords:** Multiple sclerosis, Aging, Dual-task walking, Gait variability

## Abstract

**Background:**

Greater gait variability is associated with falls in aging and multiple sclerosis. However, whether older adults with MS (OAMS), show higher gait variability relative to healthy older adults (HOA), under single and dual-task walking conditions, has not been reported. Furthermore, it is unclear whether practice may improve gait variability in both groups.

**Research question:**

Is gait variability higher in OAMS relative to HOA, particularly in DTW compared to STW? Furthermore, does practice result in decreased gait variability in both groups, notably under DTW compared to STW?

**Methods:**

We examined the effect of within-session practice on gait variability during single (STW) and dual (DTW) task gait conditions. OAMS (n = 97, mean±SD age: 65 ± 5 years, 66 females) and HOA (n = 113, mean±SD age: 68 ± 7 years, 73 females) were recruited. Practice effects on gait variability were evaluated over three repeated counterbalanced STW and DTW trials. Gait variability measures included Coefficient of Variation (CV) in stride velocity, stride length, and swing time.

**Results:**

OAMS demonstrated higher gait variability, on all measures, relative to HOA during both STW and DTW (P < 0.001). Gait variability on all measures was higher in DTW compared to STW, (P < 0.05). Practice resulted in decreased gait variability (P < 0.01) on all measures in both OAMS and HOA. Furthermore, practice resulted in decreased temporal gait variability, as measured by swing time CV, under DTW in particular (P < 0.05).

**Significance:**

In conclusion, OAMS exhibited greater gait variability than HOA, yet both groups demonstrated decreases in temporal and spatial gait variability after within-session practice, notably under DTW, which in turn may reduce fall risk.

## 1. Introduction

Multiple sclerosis (MS) is an autoimmune neurological disease characterized by diffuse axonal damage and brain atrophy that results from demyelination and neurodegeneration in the central nervous system [1,2]. There is a significant shift in the prevalence of MS amongst older adults [3], which is expected to result in co-occurring MS and aging effects on mobility and cognition. Mobility impairments are one of the most common symptoms in older adults with MS (OAMS) [4–6] and typically worsen with MS disease progression [4]. Greater gait variability, or fluctuations in gait characteristics across strides, is associated with mobility impairment and falls in MS [7]. However, whether OAMS show greater gait variability relative to healthy older adults (HOA), under single task walking (STW) and dual-task walking (DTW) conditions, has not been reported. Furthermore, it is unclear whether acute practice may improve gait variability in both groups.

Prior work suggests that gait variability is higher in MS [8]. Furthermore, increased gait variability has been associated with declines in mobility and falls in older adults [9,10], and worse cognitive processing speed in MS [11]. While other neurological populations have exhibited increased gait variability, relative to MS [8], the younger age of MS cohorts in prior work may have been a contributing factor to the less pronounced differences seen in MS. Thus, through the examination of OAMS, this study provides an important step towards furthering our understanding of the impact of MS on gait variability in older adults.

Higher gait variability have been observed during DTW, relative to STW, in both older adults with a fall history [12] and MS [13]. In particular, DTW has demonstrated higher swing time variability in older adult fallers vs. non-fallers [12]. Furthermore, higher stance time and swing time variability during STW has been associated with declines in walking function, while lower step length variability during STW has been associated with improved walking function in older adults [9]. Thus, examining changes in both temporal and spatial gait variability may provide potential outcome measures of future interventions in OAMS. Aging and MS have been found to adversely impact learning and performance [14,15]. While dual-task training in MS has not always been found to lead to improved gait function [13], recent work suggests that both OAMS and HOA may improve gait speed after an acute bout of dual-task training [16]. Thus, the examination of practice effects in this study should further our understanding of short-term benefits of dual-task training on gait variability and provide a basis for future interventions in OAMS.

The present study was designed to evaluate the effects of task (STW vs. DTW) and within-session practice on gait variability in OAMS and HOA. We hypothesized that gait variability would be higher in OAMS relative to HOA, particularly during DTW compared with STW, and that within-session practice would result in decreased gait variability in both groups, notably under DTW.

## 2. Methods

This study was approved by the IRB of Albert Einstein College of Medicine (Protocol #2019–10049). All participants reviewed and signed a written informed consent form in the first study visit. This study has been executed in adherence with The Code of Ethics of the World Medical Association (Declaration of Helsinki).

### 2.1. Participants

OAMS (n = 97, mean age= 66 years, female=66) and HOA (n = 113, mean age= 68 years, female=73) enrolled in an ongoing study titled “Brain Predictors of Mobility and Falls in Older Adults with Multiple Sclerosis” [17]. OAMS participants were mostly with relapsing-remitting (n = 59) or secondary progressive (n = 22), while a smaller number were with primary progressive (n = 7) or an undetermined (n = 9) MS subtype (Table 1). Disability was quantified using the Patient Determined Disease Steps (PDDS), a validated, self-reported ordinal scale (range=0–8)[18]. Inclusion criteria for OAMS included physician-confirmed MS diagnosis, determined using the revised McDonald criteria [19], and at least 6 months on a stable disease-modifying-therapy prior to study. For both cohorts, diagnosis of any major neurological, psychiatric, or medical disease (excluding MS), inability to ambulate independently, contraindication to MRI, impairment of vision or hearing that would negatively impact testing served as exclusion criteria.

### 2.2. Procedures

OAMS participants were recruited from regional treatment centers and patient registry lists. Both OAMS and HOA cohorts were screened using a structured telephone interview to obtain verbal consent, assess medical and psychological history, and screen for dementia, mobility, and functional abilities to determine study eligibility. Following completion of the telephone interview, participants were scheduled for two in-person study visits in the medical center. The first visit consisted of a battery of neuropsychological tests, mobility protocols including the combined burst measurement, DTW paradigm, and questionnaires. The second visit included an MRI of the brain without contrast and additional questionnaires. Participants were asked to complete three trials of STW and DTW tasks to determine the influence of short-term practice effects. In the STW, participants were asked to walk on the electronic walkway (20ftx4ft) at their “normal pace” for three consecutive loops in each trial, without any time constraints. In DTW, participants were asked to walk around the walkway for three consecutive loops at their normal pace while reciting every other letter of the alphabet. This task was selected given its widespread use and validation in measuring motor, cognitive, and dual-task performance in MS [20]. Participants started reciting every other letter with the letter A or B, which was randomized across DTW trials. Participants were instructed to pay equal attention to both tasks. In each trial, task conditions were presented in a counterbalanced order using a Latin-square design. Reliability and validity for this walking paradigm have been previously established [21]. Participants received a 5-minute break between each repeated trial.

### 2.3. Gait variability measures

An instrumented walkway using ProtoKinetics Movement Analysis Software was used to measure quantitative gait variability parameters during STW and DTW (Zenometrics, LLC; Peekskill, NY). We removed the turning components of each walking trial and kept data on just the straight-line walking portions, which provided a similar length as five-to-six trials of clinical evaluations of gait in MS, such as the timed 25-foot walk [22]. Coefficient of Variation (CV) was calculated by dividing the standard deviation by the mean. Stride velocity CV (%), stride length CV (%), and swing time CV (%) during walking were used as the outcome measures in this study. Reliability of CV measures in MS have been demonstrated to be moderate to excellent during single and DTW [23].

### 2.4. Covariates

Age, sex, years of education, and global health status (GHS) served as covariates to account for possible effects of confounders and overall cognition on gait performance during STW and DTW. The GHS, a comorbidity measure, computes a total score based on the presence/absence of the following clinical conditions: diabetes, chronic heart failure, arthritis, hypertension, depression, stroke, Parkinson’s disease, chronic obstructive lung disease, angina, and myocardial infarction score (range 0–10) [24].

### 2.5. Statistical analysis

**Fig. 1.**
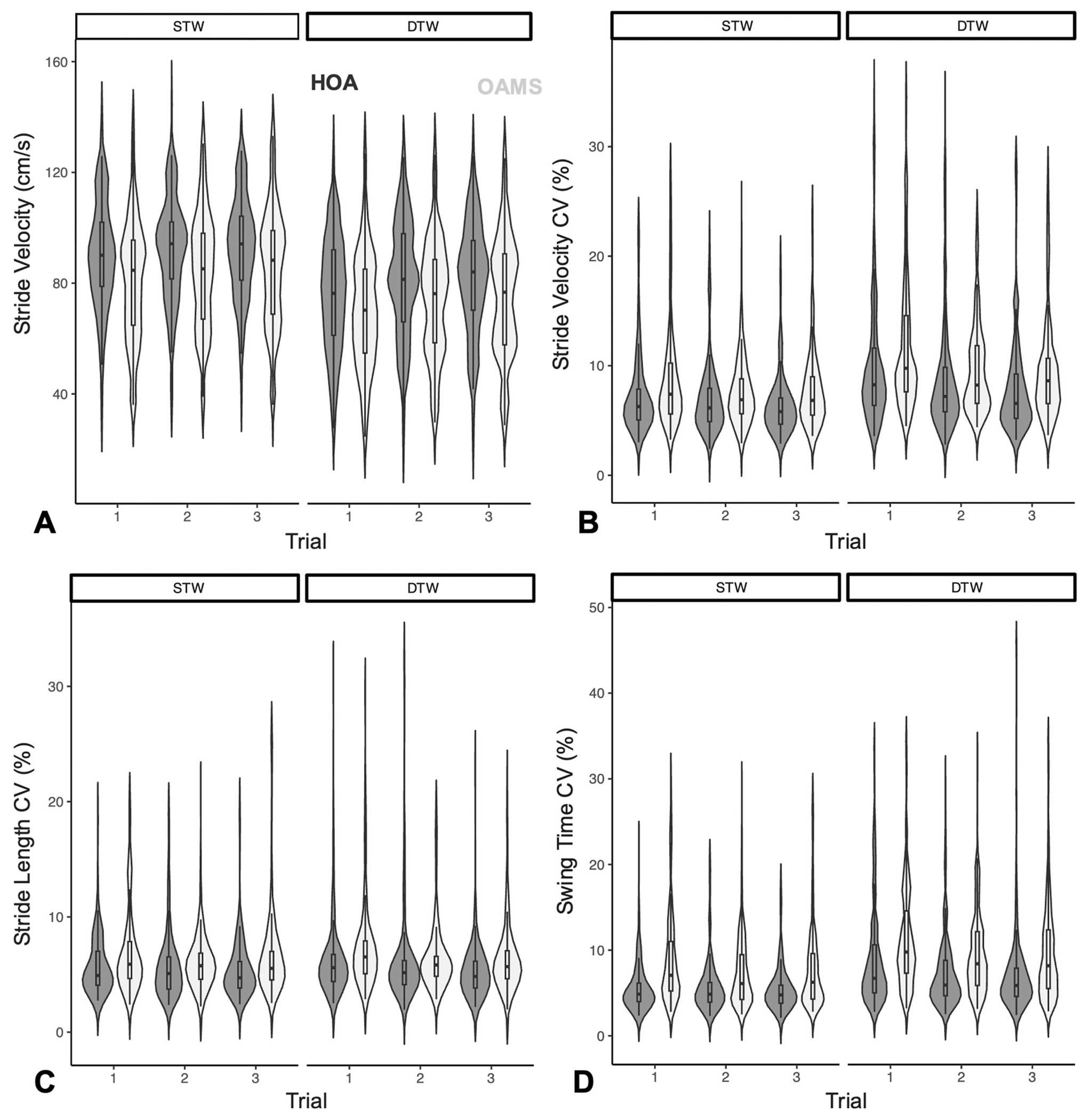
Violin plots of raw A) stride velocity, B) stride velocity CV, C) stride length CV, and D) swing time CV across tasks and trials (1–3) in OAMS (light gray) and HOA (dark gray). *NOTE: STW = single-task walk; DTW = dual-task walk; HOA = healthy older adults; OAMS = older adults with multiple sclerosis*.

**Fig. 2.**
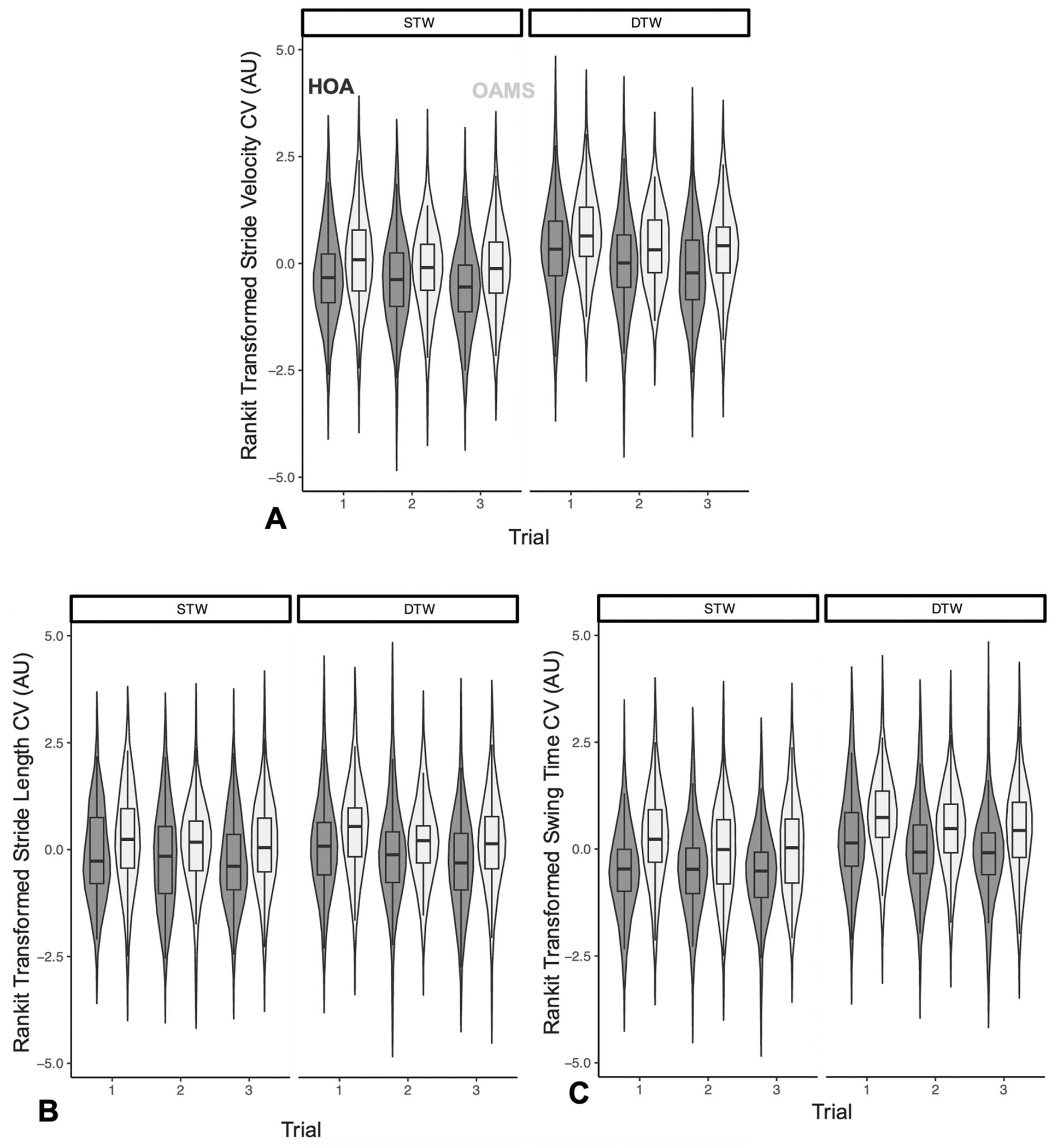
Violin plots of rankit-transformed A) stride velocity CV, B) stride length CV, and C) swing time CV across tasks and trials (1–3) in OAMS (light gray) and HOA (dark gray). *NOTE: STW = single-task walk; DTW = dual-task walk; HOA = healthy older adults; OAMS = older adults with multiple sclerosis*.

Descriptive statistics were used to summarize all demographic characteristics (mean±SD for continuous measures, count and percent for categorical variables) and tabulated per group. Differences in gait variability measures between OAMS and HOA were determined using an independent sample *t*-test or chi-square test. We used full-factorial linear mixed effects models (LMMs), which account for correlations among measurements within the same participant to compare stride velocity CV, stride length CV, and swing time CV between OAMS and HOA during STW and DTW, and across trials. Task and trial conditions served as within-subject repeated measures, and group status as a two-level between-subject variable. In all models, STW served as the reference task condition; trial 1 served as a reference for other trials to evaluate practice effects. Analyses were first run unadjusted and then adjusted for covariates. To examine moderation effects, two-way interactions of task-by-trial, group-by-task, and group-by-trial were conducted as well as three-way interactions of group-by-task-by-trial. In all LMMs, random intercept and unstructured covariance type were used, fixed effects of all variables of interest and their interactions were tabulated. This study focused on testing two primary hypotheses across three outcome measures, but as each full factorial model resulted in 16 linear contrasts of individual effects, Hochberg’s method [25] for controlling multiple comparisons was used to highlight key differences. Analyses were adjusted for covariates and were run with rankit-transformed [26] variables (Fig. 1). Analyses using transformed data are presented in this study for all variables (Fig. 2). As a secondary analysis, associations between raw gait variability measures and age, and between raw gait variability measures and stride velocity were examined using Pearson’s correlation (See Supplementary Materials). Tests of statistical significance were two-sided, and p-value< .05 was considered statistically significant. Data were analyzed using R (version 4.3.1) statistical software [27].

## 3. Results

### 3.1. Participant characteristics

Participant characteristics were tabulated per group (Table 1), while mean (SD) gait performance and gait variability stratified by trial is provided in Supplementary Table S1. There were more females in OAMS (68 %) compared to HOA (65 %) group (p < 0.001). Mean age and years of education were lower in OAMS (65.0 ± 4.7 years, 15.1 ± 2.4 years of education) compared to HOA (68.0 ± 7.2 years, 16.3 ± 2.4 years of education) group (p < 0.001). Mean GHS was comparable in OAMS (1.3 ± 1.2) and HOA (1.2 ± 0.9) groups, both indicating a low level of comorbid disease in the study population (p = 0.67). Unadjusted and adjusted analyses were not materially different for all LMMs. Therefore, analyses that fully adjusted for all covariates (age, sex, education, and GHS) were presented to account for confounding factors in analyses.

### 3.2. Stride velocity variability

The overall LMM revealed statistically significant trial (F=22.55, p < 0.001), task (F=140.84, p < 0.001), group (F=25.36, p < 0.001), and age effects (F=11.38, p < 0.001) (Fig. 2A). Two-way and 3-way interactions were not statistically significant. Contrasts of task effects revealed significantly higher rankit-transformed stride velocity CV in DTW compared to STW (p < 0.001). Group effects revealed significantly greater rankit-transformed stride velocity CV in OAMS compared to HOA (p < 0.001). Rankit-transformed stride velocity CV demonstrated significant increases with age (p < 0.001). A detailed summary of this model is presented in Table 2.

### 3.3. Stride length variability

The overall LMM revealed significant trial (F=8.54, p < 0.001), task (F=6.33, p = 0.012), group (F=22.86, p < 0.001), sex (F=7.83, p = 0.006), and age effects (F=8.41, p = 0.004) (Fig. 2B). Two-way and 3-way interactions were not statistically significant. Contrasts of group effects revealed significantly greater rankit-transformed stride length CV in OAMS compared to HOA (p = 0.001). Rankit-transformed stride length CV demonstrated significant increases with age (p = 0.004). A detailed summary of this model is presented in Table 2.

### 3.4. Swing time variability

The overall LMM revealed significant effects of trial (F=18.82, p < 0.001), task (F=228.74, p < 0.001), group (F=34.59, p < 0.001), age (F=15.77, p < 0.001), GHS (F=5.44, p = 0.021), and task-by-trial (F=3.95, p = 0.021) (Fig. 2C). No other two-way and 3-way interactions were statistically significant. Contrasts of individual effects revealed significantly higher rankit-transformed swing time CV in DTW compared to STW (p < 0.001). Group effects revealed significantly higher rankit-transformed swing time CV in OAMS compared to HOA (p < 0.001). Two-way task-by-trial interactions revealed significantly larger declines in rankit-transformed swing time CV in DTW, compared to STW, when going from trial 1 to trial 3 (p = 0.002). Rankit-transformed swing time CV demonstrated significant increases with age (p < 0.001). A detailed summary of this model is presented in Table 2.

## 4. Discussion

This study examined the effect of within-session practice on gait variability under single and dual-task conditions in OAMS and HOA. Partly consistent with our primary hypothesis, OAMS exhibited higher gait variability, relative to HOA. Consistent with our second hypothesis, gait variability was greater in DTW compared to STW likely due to higher cognitive demands of the former task condition. Further, both OAMS and HOA exhibited greater practice-related declines in gait variability, as defined by swing time variability, under DTW in comparison to STW. This finding can be interpreted as a larger benefit from within-session practice in DTW tasks, which may be due to the novelty of the task or change in prioritization of the task. Lastly, the larger improvements in swing time variability during DTW tasks may be partly due to the addition of a cognitive task and subsequent increase in gait variability that may be more sensitive to practice effects.

### 4.1. Effect of MS in gait variability of older adults

Consistent with prior work in MS, this study found gait variability to be higher in OAMS, compared to HOA [28–30]. A prior review [8] of gait variability in adults with neurological disorders, had demonstrated a gap in stride-based measures and examination of OAMS. Thus, the use of a reliable and valid [21] walking paradigm in OAMS presents an opportunity to examine the coupling effects of age-related and MS-related changes on gait variability. In particular, spatiotemporal, temporal and spatial gait variability, were demonstrated to be higher in OAMS, relative to HOA, in this study, consistent with impaired gait rhythmicity [8]. As seen from the mean and SD of gait performance and gait variability measures on Supplementary Table S1, OAMS had both a lower mean stride velocity and a higher SD across task and trials, suggesting that the higher CV seen in OAMS, relative to HOA, is not entirely due to a decreased stride velocity. Lastly, while prior findings suggest a minimum clinically important difference of 0.01 s for stance time and swing time variability and 0.25 cm for step length variability [31], we were unable to find similar guidance for establishing clinical significance using the outcome measures in this study. Thus, future work is needed to establish the clinical significance of the observed changes.

### 4.2. Effect of practice in older adults with and without MS

While overall, a significant trial effect was observed in all outcome measures, these changes were primarily driven by decreases in swing time variability, with practice in DTW, as demonstrated by a significant two-way interaction of trial-by-task for swing CV only. We fnd that practice-related improvements in swing time variability in trial 3, relative to trial 1, are suggestive of implicit learning that could be targeted in future neurorehabilitation, consistent with prior work [32]. Given the improved stride velocity performance across trials in DTW, as seen in Fig. 1 and Supplementary Table S1, and the improved cognitive performance previously seen in the number of correct utterances per minute seen in a prior study of this cohort [16], the improvement in gait variability in the DTW task does not seem to be due to a shift of focus from the cognitive to the motor task.

To our knowledge, this is the first study demonstrating within-session improvements in gait variability while DTW in OAMS. The ability of OAMS and HOA to decrease gait variability within a single session is significant, despite the differences due to aging and MS on structural and functional brain networks related to mobility [33], as it bolsters the evidence for dual-task training as a promising potential avenue for neurorehabilitation in not only older adults, but also OAMS.

### 4.3. Associations of age and performance with gait variability

Gait variability, and particularly stride velocity CV, demonstrated stronger associations with age during STW, rather than DTW, in both OAMS and HOA. These findings are consistent with prior findings of associations between gait variability and age [34]. Age-related associations with gait variability may be partly driven by functional status and cognition changes, given prior associations observed between gait variability and functional status [35] and cognition [36] in older adults. Given the associations observed between structural brain changes and temporal gait variability [37], further inquiry into the association of structural brain changes with spatiotemporal gait variability in OAMS may be beneficial for identifying neural mechanisms underlying changes in gait variability.

Spatiotemporal and temporal gait variability measures demonstrated strong associations with stride velocity in both STW and DTW, in both OAMS and HOA. Given the similar range of stride velocities observed in both groups in either STW or DTW tasks from these correlations and performance differences seen between groups in supplementary materials, these findings suggest that increases seen in stride velocity CV are not entirely driven by gait performance differences.

### 4.4. Study strengths, limitations, and future directions

This study furthers extant research by providing novel insights into the combined and possibly synergistic effects of aging and MS on gait variability. This study suggests that even in short time courses and a single session, gait variability may improve in both OAMS and HOA. Furthermore, gait variability improvements have important implications for fall interventions, as falls are common in OAMS [38,39] and present a major individual and public health concern. Participants were well-characterized, but due to study design and eligibility criteria requiring ambulatory capabilities, relatively intact cognition, and no contraindications or aversion to MRI, generalizability of the findings to more variable and impaired samples should be evaluated in future work. Given the importance of MS disability and disease progression on mobility, future work should examine their association with gait variability. While analysis controlled for potential confounders, the unweighted summation of 10 diseases used to derive the GHS variable provided limited adjustment for comorbidity in the current study. Future work should examine the association between gait variability and falls in OAMS with either relapsing-remitting or progressive subtypes, given differences observed in predictors of prospective falls [38,39]. Furthermore, future work should also examine the association between gait variability and brain function in OAMS given the links observed between gait variability and brain function, including variability in hemodynamic responses [40]. Lastly, the acute practice effects on gait variability outcomes seen in STW and DTW tasks should be examined in longer time intervals in future work, to evaluate the implications on motor learning and retention of benefits in OAMS.

## 5. Conclusion

In summary, this study provided the first evidence that gait variability is higher in OAMS, relative to HOA, yet both groups demonstrated decreases in temporal and spatial gait variability after within-session practice, particularly during DTW, which in turn may reduce fall risk. These findings suggest that practice may improve gait variability outcomes in OAMS and HOA and may benefit from targeted approaches aimed at individuals with increased gait variability, which merit further examination.

## Supplementary Material

supplementary material

## Figures and Tables

**Table 1:** Sample characteristics stratified by group.

Variable	OAMS (*n = 97*)		HOA (*n = 113*)		p value

	Mean	SD	Mean	SD	
Age (years)	65.0	4.7	68.0	7.2	< 0.001
Education (years)	15.1	2.4	16.3	2.4	< 0.001
GHS (0–10)	1.3	1.2	1.2	0.9	0.67
PDDS (0–8)	2.1	1.8			
	Number	Percent	Number	Percent	
Females	66	68.0	73	64.6	< 0.001
MS subtypes					
Relapse-Remitting	59	60.8			
Secondary Progressive	22	22.7			
Primary Progressive	7	7.2			
Undetermined	9	9.3			

NOTE: GHS = global health status; PDDS = patient determined disease steps; OAMS = older adults with multiple sclerosis; HOA = healthy older adults. Independent t-tests were used for group differences in continuous variables. Chi-square test was used for group difference in females.

**Table 2:** Linear mixed effects models for outcome measures.

	Variable	Estimate	SE	p value	95 % CI lower	95 % CI upper
Rankit Transformed Stride Velocity CV	Intercept	−2.455	0.649	**< 0.001**	−3.715	−1.196
	DTW vs. STW	0.661	0.102	**< 0.001**	0.462	0.861
	Trial 2 vs. Trial 1	−0.098	0.099	0.325	−0.292	0.096
	Trial 3 vs. Trial 1	−0.281	0.101	0.005	−0.477	−0.084
	OAMS vs. HOA	0.500	0.137	**< 0.001**	0.234	0.766
	Sex	0.048	0.100	0.634	−0.146	0.242
	Age (years)	0.027	0.008	**< 0.001**	0.011	0.042
	Education (years)	0.016	0.019	0.400	−0.021	0.054
	GHS	0.050	0.044	0.256	−0.035	0.136
	DTW vs. STW*Trial 2 vs. Trial 1	−0.235	0.142	0.098	−0.511	0.042
	DTW vs. STW*Trial 3 vs. Trial 1	−0.252	0.143	0.078	−0.531	0.027
	DTW vs. STW*OAMS vs. HOA	−0.066	0.149	0.660	−0.356	0.225
	Trial 2 vs. Trial 1 *OAMS vs. HOA	−0.117	0.147	0.426	−0.404	0.170
	Trial 3 vs. Trial 1 *OAMS vs. HOA	0.079	0.148	0.593	−0.210	0.368
	DTW vs. STW*Trial 2 vs. Trial 1 *OAMS vs. HOA	0.145	0.208	0.487	−0.262	0.552
	DTW vs. STW*Trial 3 vs. Trial 1 *OAMS vs. HOA	0.085	0.210	0.686	−0.324	0.494
Rankit Transformed Stride Length CV	Intercept	−2.213	0.652	**< 0.001**	−3.477	−0.949
	DTW vs. STW	0.221	0.109	0.042	0.009	0.433
	Trial 2 vs. Trial 1	−0.075	0.106	0.480	−0.281	0.131
	Trial 3 vs. Trial 1	−0.139	0.107	0.195	−0.348	0.070
	OAMS vs. HOA	0.456	0.141	**0.001**	0.182	0.731
	Sex	0.281	0.100	0.006	0.086	0.476
	Age (years)	0.020	0.008	**0.004**	0.008	0.039
	Education (years)	0.020	0.019	0.315	−0.018	0.057
	GHS	0.063	0.044	0.152	−0.022	0.149
	DTW vs. STW*Trial 2 vs. Trial 1	−0.103	0.151	0.493	−0.397	0.191
	DTW vs. STW*Trial 3 vs. Trial 1	−0.233	0.152	0.126	−0.529	0.064
	DTW vs. STW*OAMS vs. HOA	0.033	0.158	0.834	−0.276	0.343
	Trial 2 vs. Trial 1 *OAMS vs. HOA	−0.044	0.156	0.779	−0.349	0.261
	Trial 3 vs. Trial 1 *OAMS vs. HOA	0.067	0.158	0.670	−0.240	0.375
	DTW vs. STW*Trial 2 vs. Trial 1 *OAMS vs. HOA	−0.058	0.222	0.794	−0.491	0.375
	DTW vs. STW*Trial 3 vs. Trial 1 *OAMS vs. HOA	−0.011	0.223	0.961	−0.446	0.424
Rankit Transformed Swing Time CV	Intercept	−3.048	0.728	**< 0.001**	−4.460	−1.635
	DTW vs. STW	0.791	0.082	**< 0.001**	0.632	0.951
	Trial 2 vs. Trial 1	0.031	0.080	0.698	−0.124	0.186
	Trial 3 vs. Trial 1	−0.051	0.081	0.528	−0.208	0.107
	OAMS vs. HOA	0.854	0.134	**< 0.001**	0.594	1.114
	Sex	0.077	0.113	0.493	−0.141	0.296
	Age (years)	0.035	0.009	**< 0.001**	0.018	0.053
	Education (years)	−0.003	0.022	0.900	−0.045	0.040
	GHS	0.116	0.050	0.021	0.019	0.212
	DTW vs. STW*Trial 2 vs. Trial 1	−0.318	0.113	0.005	−0.539	−0.097
	DTW vs. STW*Trial 3 vs. Trial 1	−0.357	0.114	**0.002**	−0.580	−0.133
	DTW vs. STW*OAMS vs. HOA	−0.300	0.119	0.012	−0.532	−0.067
	Trial 2 vs. Trial 1 *OAMS vs. HOA	−0.315	0.118	0.008	−0.545	−0.086
	Trial 3 vs. Trial 1 *OAMS vs. HOA	−0.150	0.119	0.207	−0.382	0.082
	DTW vs. STW*Trial 2 vs. Trial 1 *OAMS vs. HOA	0.341	0.167	0.041	0.016	0.667
	DTW vs. STW*Trial 3 vs. Trial 1 *OAMS vs. HOA	0.248	0.168	0.140	−0.080	0.575

NOTE: STW = single task walk; DTW = dual task walk; bold text indicates significant results after applying Hochberg’s method for controlling for multiple comparisons (p < 0.0042).
