# Supplementary material for: Within-session dual-task walking practice improves gait variability in older adults with multiple sclerosis
Source: Gait Posture. Author manuscript; Available in PMC 2026 May 18. (PMC12048238; doi:10.1016/j.gaitpost.2025.03.011)

**Supplementary Results.**

*Associations of age and performance with gait variability*

First, higher stride velocity CV was associated with increased age (r^2^ = 0.009, p<0.001), and higher swing time CV was associated with increased age (r^2^ = 0.008, p=0.002). Further breakdown by task, demonstrated that higher STW stride velocity CV was associated with increased age (r^2^ = 0.0256, p<0.001), as well as higher STW swing time CV (r^2^ = 0.011 p=0.011), and higher DTW swing time CV (r^2^ = 0.007, p=0.036). Further breakdown by group in each task demonstrated that STW stride velocity CV associations with age were driven by both OAMS (r^2^ = 0.020, p=0.01867) and HOA (r^2^ = 0.083, p<0.001), while STW and DTW swing time CV associations with age were driven by HOA (r^2^ = 0.110, p<0.001 in STW; r^2^ = 0.048, p<0.001 in DTW) (Supplementary Figure 1). No further statistically significant associations were observed.

Overall, significant correlations were found between all outcome measures and stride velocity (p < 0.001). Lower stride velocity CV (r^2^ = 0.260), stride length CV (r^2^ = 0.011), and swing time CV (r^2^ = 0.408) were associated with higher stride velocity. Further breakdown by task, demonstrated that lower STW stride velocity CV (r^2^ = 0.233, p<0.001) and STW swing time CV (r^2^ = 0.385, p<0.001) were associated with higher stride velocity; while lower DTW stride velocity CV (r^2^ = 0.235, p<0.001), DTW stride length CV (r^2^ = 0.013, p=0.004), and DTW swing time CV (r^2^ = 0.387, p<0.001) were associated with higher stride velocity (Figure 3 and Supplementary Figure 1). Further breakdown by group in each task demonstrated that STW and DTW stride velocity CV and swing time CV associations with stride velocity were driven by both OAMS and HOA (r^2^ = 0.157 – 0.400, p<0.001), while DTW stride length CV was driven by HOA (r^2^ = 0.042, p<0.001) (Supplementary Figures 1 and 2) No further statistically significant associations were observed.

**Supplementary Table S1.** Performance and gait variability in HOA and OAMS stratified by trial.

*NOTE: STW = single task walk; DTW= dual task walk; OAMS = older adults with multiple sclerosis; HOA = healthy older adults.*

**Supplementary Figure 1:** Associations of A) stride velocity CV with age, B) stride velocity CV with stride velocity, C) swing time CV with age, and D) swing time CV with stride velocity across tasks in OAMS (light gray) and HOA (dark gray). *NOTE: STW = single task walk; DTW = dual task walk; HOA = healthy older adults; OAMS = older adults with multiple sclerosis.*


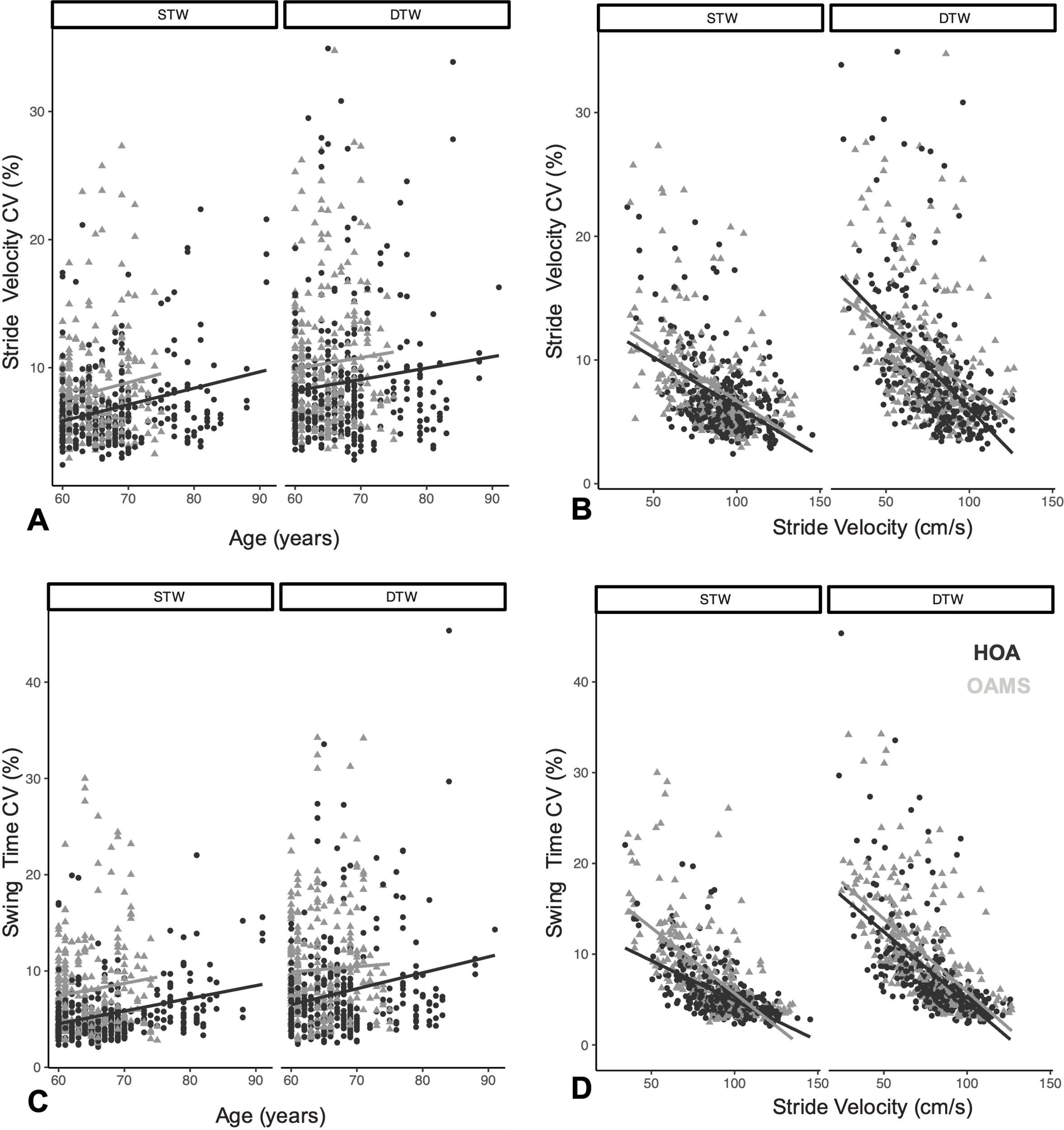


**Supplementary Figure 2:** Associations of stride length CV with stride velocity across tasks in OAMS (light gray) and HOA (dark gray). *NOTE: STW = single task walk; DTW = dual task walk; HOA = healthy older adults; OAMS = older adults with multiple sclerosis.*


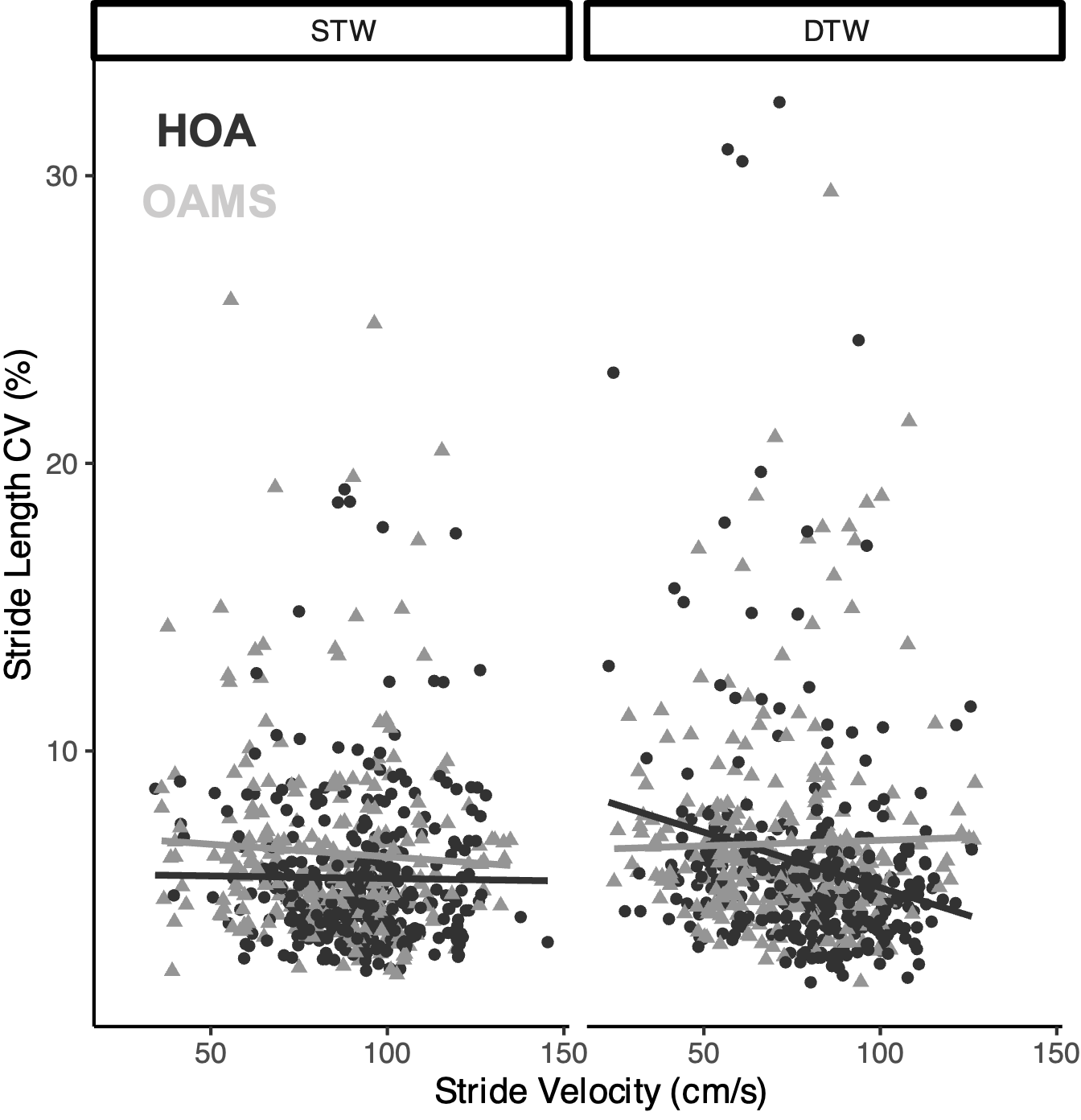

Supplement: supplementary material [file NIHMS2072260-supplement-supplementary_material.docx]
